# Butyrate Permeation across the Isolated Ovine Reticulum Epithelium

**DOI:** 10.3390/ani10122198

**Published:** 2020-11-24

**Authors:** Reiko Rackwitz, Franziska Dengler, Gotthold Gäbel

**Affiliations:** Institute of Veterinary Physiology, University of Leipzig, 04103 Leipzig, Germany; dengler@vmf.uni-leipzig.de (F.D.); gaebel@rz.uni-leipzig.de (G.G.)

**Keywords:** short-chain fatty acids (SCFA), transepithelial transport, MCT1, NHE, Na^+^/K^+^-ATPase, reticulorumen, Ussing

## Abstract

**Simple Summary:**

Short-chain fatty acids are the main source of energy for ruminants. The effective uptake of these substrates from the forestomach is a prerequisite for the health and performance of these animals. Thus far, the mechanisms of uptake have been investigated almost exclusively in the epithelium of the largest forestomach section, the rumen. Previous research suggests that the reticulum is also involved in the uptake of short-chain fatty acids, but the mechanisms involved have not been studied and may differ from those known from the rumen epithelium due to the different milieu in this compartment. To investigate this, ovine reticulum epithelium was mounted in Ussing chambers, and the transport of radiolabeled butyrate (as a representative of short-chain fatty acids) across the tissue was measured with and without the addition of inhibitors of particular transport proteins. Our results show that butyrate can be taken up effectively across the reticulum epithelium via pathways that are energized by the Na^+^/K^+^-ATPase and may involve monocarboxylate transporters, sodium-proton exchangers, and anion channels. However, our results are not completely congruent to those obtained in the rumen epithelium. These modifications could assure the effective uptake of short-chain fatty acids from the reticulum lumen under the particular conditions (p. e. high pH) of this forestomach compartment.

**Abstract:**

We hypothesized that, due to the high pH of this compartment, the reticulum epithelium displays particular features in the transport of short-chain fatty acids (SCFA). Ovine reticulum epithelium was incubated in Ussing chambers using a bicarbonate-free buffer solution containing butyrate (20 mmol L^−1^). p-hydroxymercuribenzoic acid (pHMB), 5-(N-Ethyl-N-isopropyl)amiloride (EIPA), or ouabain were added to the buffer solution as inhibitors of monocarboxylate transporters, sodium-proton-exchangers, or the Na^+^/K^+^-ATPase, respectively. The short-circuit current (I_sc_) and transepithelial conductance (G_t_) were monitored continuously while the flux rates of ^14^C-labelled butyrate were measured in the mucosal-to-serosal (J_ms_^but^) or serosal-to-mucosal direction (J_sm_^but^). Under control conditions, the mean values of I_sc_ and G_t_ amounted to 2.54 ± 0.46 µEq cm^−2^ h^−1^ and 6.02 ± 3.3 mS cm^−2^, respectively. J_ms_^but^ was 2.1 ± 1.01 µmol cm^−2^ h^−1^ on average and about twice as high as J_sm_^but^. Incubation with ouabain reduced J_ms_^but^, while J_sm_^but^ was not affected. The serosal addition of EIPA did not affect J_ms_^but^ but reduced J_sm_^but^ by about 10%. The addition of pHMB to the mucosal or serosal solution reduced J_ms_^but^ but had no effect on J_sm_^but^. Mucosally applied pHMB provoked a transient increase in the I_sc_. The serosal pHMB sharply reduced I_sc_. Our results demonstrate that butyrate can be effectively transported across the reticulum epithelium. The mechanisms involved in this absorption differ from those known from the rumen epithelium.

## 1. Introduction

The reticulorumen (RR) is the most proximal and largest part of the forestomach system of ruminants. It is lined by squamous epithelium and consists of two morphological distinguishable sections, the rumen and the smaller reticulum, the latter with a characteristic surface of polygonal fields separated by crests [1]. Functionally, the RR can be considered as a fermentation chamber where long-chain carbohydrates (cellulose, hemicellulose) are broken down by microbial fermentation. The products of these processes are mainly short-chain fatty acids (SCFA) with acetate, propionate, and butyrate being the most abundant compounds [2]. SCFA are the main substrates to cover the energy requirement of ruminants [3]. Consequently, the largest part of the SCFA produced in the RR is absorbed directly from its lumen into the blood [4,5,6]. Due to the importance of this absorption for the ruminants’ nutrition, health, and performance, the underlying mechanisms are of pivotal interest. Currently, the available data suggest that a combination of diverse mechanisms is involved in this absorption, including the lipophilic diffusion of protonated SCFA [7], the exchange of SCFA anions (SCFA^−^) for bicarbonate or chloride [8,9], the proton-coupled transport of SCFA^−^ via monocarboxylate transporters (MCTs) [10,11,12], and the channel-mediated permeation of SCFA^−^ [13,14]. In addition, the export of protons out of the epithelium via Na^+^/H^+^-exchangers (NHE; [15]) and the supply of bicarbonate by Na^+^-HCO_3_^−^ cotransporters [16] support the mechanisms stated above. This diversity of mechanisms involved in SCFA absorption protects the pH homeostasis of the epithelium while ensuring an adequate energy supply for the animal under different feeding conditions and/or during alterations of the milieu in the RR.

However, the in vitro studies underlying these conclusions were conducted almost exclusively on the rumen epithelium or ruminal epithelial cells. Few studies are available concerning the transport properties of the reticulum epithelium, and none of these cover the issue of SCFA absorption. Additionally, studies using the washed and isolated RR in vivo cannot discriminate between the two compartments, rumen and reticulum.

Nevertheless, besides its particular macroscopic structure [1], the reticulum epithelium has been shown to differ from ruminal epithelium in displaying a higher Na^+^/K^+^-ATPase activity [17] as well as a higher short-circuit current (I_sc_), combined with a remarkably higher transepithelial conductance (G_t_) [18]. Based on these data, a generally higher transport activity of the reticulum epithelium in comparison to the ruminal epithelium can be assumed. Furthermore, the pH in the reticulum is constantly higher than that in the rumen (by 0.2–0.4 units; [19,20,21]), shifting the equilibrium between SCFA^−^ and protonated SCFA towards the anions. Under these conditions, a high rate of protein-mediated transport of SCFA^−^ can be expected. Thus, the pathways known for SCFA absorption from the rumen may also be functional in the reticulum epithelium, but potentially in a modified manner.

In the present study, we characterize selected (bicarbonate-independent) mechanisms for the transepithelial transport of butyrate across the isolated reticulum epithelium for the first time.

## 2. Materials and Methods

### 2.1. Chemicals and Buffer Solutions

The chemicals used were purchased from Sigma-Aldrich (Schnelldorf, Germany) or Carl Roth (Karlsruhe, Germany). ^14^C-labelled butyrate was obtained from Hartmann Analytic (Braunschweig, Germany). The gasses were procured from Linde Gas (Leipzig, Germany).

All the buffer solutions were bicarbonate-free, gassed with oxygen, and adjusted to pH 7.4 with gluconic acid or NaOH, respectively. The endogenous production of bicarbonate was prevented by adding ethoxyzolamide to the buffer solution (50 µmol l^−1^, [22])

The buffer solution used to rinse and transport the epithelia to the laboratory (“transport buffer”) contained (in mmol L^−1^) 125 Na^+^, 5.5 K^+^, 1.25 Ca^2+^, 1.25 Mg^2+^, 128 Cl^−^, 0.6 H_2_PO_4_^−^, 2.4 HPO_4_^2−^, 1 L-glutamine, 10 HEPES, and 10 glucose. The buffer solution used to incubate the epithelia in the Ussing chambers was assembled the same way, apart from the substitution of 20 mmol L^−1^ of chloride for the same amount of butyrate.

To inhibit MCTs, p-hydroxymercuribenzoic acid (pHMB) was applied at a final concentration of 1.6 mmol L^−1^ to the mucosal or serosal buffer solution. 5-(N-Ethyl-N-isopropyl)amiloride (EIPA) was used at a concentration of 50 µmol L^−1^ in order to inhibit NHEs. The Na^+^/K^+^-ATPase inhibitor ouabain (100 µmol l^−1^) was added to the serosal buffer solution exclusively.

### 2.2. Animals and Ethical Approval

All the experiments of this study were in accordance with the German legislation on the protection of animals and the EU directive 2010/63/EU for animal experiments. The studies were communicated to the Regierungspräsidium Leipzig under file number AZ 24-9162.11-01-T58/04 and T86/10.

For our experiments, reticulum tissues were obtained from adult female sheep (*Ovis aries*) of different ages and breeds. The animals were housed in a barn of the Veterinary Faculty at least two weeks prior to the experiments and fed with hay and water ad libitum.

On the day of the experiment, the sheep were sacrificed by exsanguination after captive bolt stunning. After that, the abdominal cavity was opened and the reticulum was removed. The tissue was rinsed several times in warm transport buffer solution until the solution remained clear. Thereafter, the epithelial layers were manually stripped off the muscle layers and stored in 38 °C warm transport buffer solution until use.

### 2.3. Incubation

The reticulum epithelium was cut along the crests and mounted in Ussing chambers, so that the crests were kept out of the aperture of the chambers. The exposed area amounted to 1.0 cm^2^. Between the epithelium and the plastic halves of the Ussing chamber, silicon rubber rings were placed to minimize edge damage. On both the mucosal and the serosal side, the same buffer solution containing 20 mmol L^−1^ butyrate was applied (composition see above). The buffer solutions were kept constantly at 38 °C and stirred continuously by a gas lift system. The latter provided 100% oxygen for the oxygenation of the solutions. Experiments were started after an equilibration period of about 20 min.

### 2.4. Electrophysiology

The Ussing chambers were connected to a computer-controlled voltage clamp device (Dipl.-Ing. K. Mussler–Scientific Instruments, Aachen, Germany). The transepithelial potential difference (PD_t_) was continuously measured through agar bridges and Ag/AgCl-electrodes. Another pair of electrodes and agar bridges served to apply bipolar current pulses of 300 µA with a duration of 100 ms every 10 s. The changes in PD_t_ induced by these pulses were used to calculate the transepithelial tissue conductance (G_t_) according to Ohm’s law. Additionally, through the second pair of electrodes an external current was applied continuously to clamp the PD_t_ to 0 mV (short-circuit). This current corresponds to the current across the epithelium induced by active charge transfer (short-circuit current, I_sc_) but is directed oppositely. Before starting the experiments, the junction potentials and fluid resistance were measured and used to correct for the values obtained throughout the experiment.

Since the same buffer solution was used on the mucosal and the serosal side and short-circuit conditions were applied, there was nominally no electrochemical gradient between the mucosal and the serosal side.

Epithelia were assigned to the different treatments according to their G_t_ so that all the experimental groups displayed a similar mean G_t_ in the end.

### 2.5. Butyrate Flux Rates

^14^C-labelled butyrate (37 kBq/12 mL) was added to the buffer solution on either the mucosal (mucosal to serosal flux, J_ms_^but^) or serosal side (serosal to mucosal flux, J_sm_^but^). Epithelia were allowed to equilibrate with ^14^C-butyrate for 60 min to reach a steady state. Then, 800 µl samples were taken from the unlabeled side in 30 min intervals and the volume removed was replaced by the same amount of buffer solution. In the first flux period of one hour, all the epithelia were treated equally (control). After that, inhibitors (ouabain, pHMB, or EIPA, respectively) were added, as described above. To observe a possible time dependence of flux rates, in every experiment there also was a control which did not receive any inhibitor (“control” in figures). Thirty minutes later, a second flux period of one hour followed (treatment). At the beginning and the end of the experiment, a 100 µL sample was taken from the labelled side. Scintillation fluid (Aquasafe 300+^®^, Zinsser Analytic, Germany) was added to the samples and the radioactivity was measured in a scintillation counter (Tri Carb 2810 TR, Perkin Elmer Inc., Waltham, MA, USA) in decays per minute (dpm). Flux rates were calculated by simple ratio equation using the concentration of butyrate, the dpm of the sample from the unlabeled side, and the averaged dpm of the two samples from the labelled side.

### 2.6. Statistics

Due to the restricted availability of tissue, different numbers of epithelia were used for the particular treatments (n) per animal (N). In the case of n > 1, the respective data were pooled for each animal. Hence, statistics are based on the number of animals used. For the flux rates, every N is shown. Electrophysiological data are given as arithmetic means with their respective standard deviation (SD). Statistical testing and the display of results was carried out using the SigmaPlot 14 software package (Systat Software Inc., San Jose, CA, USA). To identify differences between two groups, paired Student’s t-test was used. The normal distribution of the data was checked automatically by the algorithms of the software used. Differences between treatments and control are referred to as statistically significant when *p* < 0.05.

## 3. Results

### 3.1. Control Conditions

**Figure 1:**
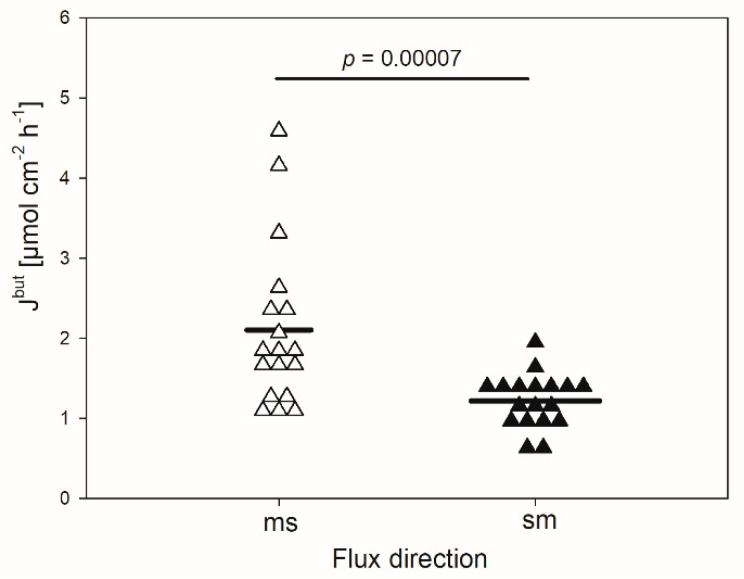
Flux rates of butyrate (J^but^) across the isolated reticulum epithelium in the mucosal to serosal (ms) and serosal to mucosal direction (sm). Each data point shown represents one animal. Solid lines represent the mean of the data (paired Student’s t-test; N = 18).

In the absence of any inhibitor, the mean flux rate of butyrate in the mucosal to serosal direction (J_ms_^but^) amounted to 2.1 ± 1.01 µmol cm^−2^ h^−1^ (Figure 1). It was significantly higher than the flux rate in the opposite direction (J_sm_^but^, 1.2 ± 0.34 µmol cm^−2^ h^−1^, *p* < 0.001). Hence, a net absorption of butyrate was observed under the conditions applied, ruling out transport by simple diffusion.

**Figure 2:**
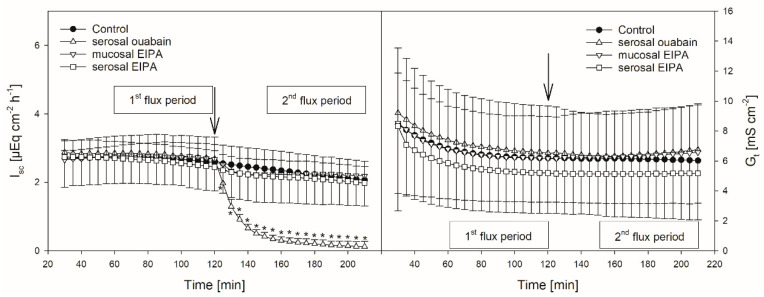
I_sc_ (left) and G_t_ (right) over time in the presence or absence of EIPA or ouabain, respectively. Arrows mark the time of inhibitor application. Asterisks indicate values significantly different from the control. Boxes indicate the two flux periods corresponding to Figure 3 (mean ± SD; paired Student’s t-test; N = 8).

**Figure 3:**
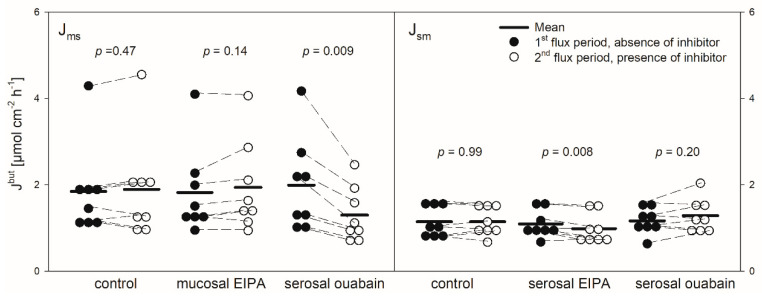
J_ms_^but^ (left) and J_sm_^but^ (right) of butyrate across the reticulum epithelium. Flux rates were measured for one hour without inhibitors in the buffer solutions (1st flux period). Then, inhibitors were added to the mucosal or serosal buffer solution as displayed. 30 min later, flux rates were measured for another hour (2nd flux period). Each data point represents one animal. Solid horizontal lines indicate the respective mean value of the group. Dashed lines connect corresponding data points of the same animal (paired Student’s t-test; N = 8).

**Figure 4:**
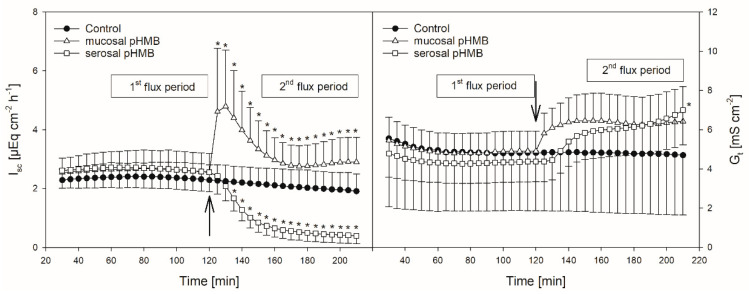
Course of I_sc_ (left) and G_t_ (right) over time in the presence or absence of pHMB in the mucosal or serosal buffer solution. Arrows indicate the time when pHMB was applied. Asterisks mark values that differ from control with *p* < 0.05. Boxes visualize the two flux periods, corresponding to Figure 5 (mean ± SD; N = 6; paired Student’s t-test).

**Figure 5:**
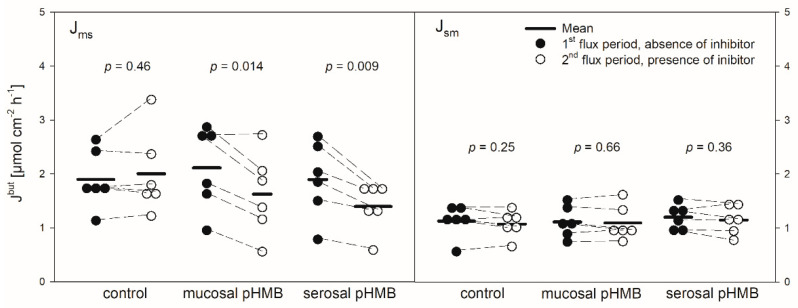
J_ms_^but^ (left) and J_sm_^but^ (right) across the reticulum epithelium in the presence or absence of pHMB in the mucosal or serosal buffer solution. Flux rates were measured for one hour in the absence of pHMB (1st flux period). Then, pHMB was added to the mucosal or serosal buffer solution. After 30 min incubation with the inhibitor, the flux rates were measured for another hour (2nd flux period). Each data point represents one animal. Solid horizontal lines indicate the mean value of the group. Dashed lines connect the corresponding data points of the same animal (paired Student’s t-test; N = 6).

The mean I_sc_ amounted to 2.54 ± 0.46 µEq cm^−2^ h^−1^. The mean G_t_ was 6.02 ± 3.3 mS cm^−2^. Only small changes in the I_sc_ and G_t_ over time could be observed (Figure 2, Figure 3, Figure 4 and Figure 5, Table 1).

### 3.2. pHMB

pHMB was used as an inhibitor of MCTs [11,12]. Thus, its addition should result in a decreased flux rate if MCTs play a role in butyrate transport across the reticulum epithelium. The application of pHMB to the mucosal or serosal side actually reduced J_ms_^but^ significantly by about 25% (Figure 5). J_sm_^but^, however, was not altered in the presence of pHMB in the mucosal or serosal buffer solution.

In comparison to control, the addition of pHMB to the mucosal side provoked a transient increase in the I_sc_ (maximum at 5.04 ± 2.05 µEq cm^−2^ h^−1^) while the serosal addition of the inhibitor led to a significant decrease in the I_sc_ (Figure 4, Table 1). Both the mucosal and serosal application of pHMB led to an increased G_t_. Though this increase was apparent, only the last measurement of G_t_ after the serosal addition of pHMB differed significantly from the control (Figure 4). However, under these conditions the difference in the mean Gt between the first and the second flux period (ΔG_t_) was significantly higher than in the control group—i.e., without the pHMB addition (Table 1).

### 3.3. EIPA

Protons that enter the cytosol by the lipophilic diffusion of SCFA or by proton-coupled transport may be extruded by NHEs. To investigate if this mechanism is involved in butyrate transport across the reticulum epithelium, the NHE inhibitor EIPA was applied to the buffer solution on the radioactively labelled side exclusively. The inhibitor had no significant effect on the electrophysiological parameters I_sc_ and G_t_ (Figure 2). Additionally, the application of EIPA to the mucosal side had no influence on J_ms_^but^ (Figure 3). However, the addition of the inhibitor to the serosal buffer solution resulted in a significant decrease in the J_sm_^but^. Nevertheless, this effect was small (−0.11 ± 0.07 µmol cm^−2^ h^−1^, ~10%, Figure 3).

### 3.4. Ouabain

Ouabain was applied to the serosal side exclusively to inhibit the Na^+^/K^+^-ATPase. After the addition of ouabain, a strong decrease in the I_sc_ could be observed, while the G_t_ remained unaffected (Figure 2). After ouabain treatment, J_ms_^but^ was reduced by 0.69 ± 0.51 µmol cm^−2^ h^−1^ (~32%) but J_sm_^but^ was not affected by the application of the inhibitor (Figure 3).

## 4. Discussion

The effective absorption of SCFA from the forestomach is crucial for covering the ruminant’s energy demand and the maintenance of the milieu in the reticulorumen [4,23,24]. While numerous in vitro studies address the pathways of SCFA absorption across the rumen epithelium, the reticulum has been neglected in this regard so far. Furthermore, in vivo studies can hardly discriminate between these two compartments. Hence, statements concerning the pathways of SCFA permeation across the reticulum epithelium are mostly deduced from findings in the rumen epithelium.

In the present study, we provide data regarding the transport of butyrate across the reticulum epithelium in vitro for the first time.

It has to be considered that butyrate may be metabolized almost completely to monocarboxylates (e.g., beta-hydroxybutyrate, lactate) inside the reticulum epithelium, as described for the rumen epithelium [3,25,26]. Hence, the flux of butyrate across the reticulum epithelium, as investigated in the present study, consists of two mechanisms—the uptake of butyrate into and the extrusion of butyrate or its metabolites out of the epithelium into the blood.

As shown for the rumen epithelium previously [7,27], we detected a net absorption of butyrate across the reticulum epithelium as well (Figure 1). The flux rates measured were similar to those reported for the rumen epithelium under comparable conditions [7]. However, in our experiments the values for the electrophysiological parameters I_sc_ and G_t_ were remarkably higher than those observed in the rumen epithelium in vitro [7,28]. This is congruent to observations from a study focusing on Na^+^ transport across the ovine reticulum epithelium [18]. In view of the electrophysiological parameters, one may assume that the reticulum epithelium displays a generally high transport capacity, as proposed by Schnorr et al. [17], with regard to the high activity of the Na^+^/K^+^-ATPase in this tissue. This may also be valid for the transport of SCFA. The flux rates of butyrate observed in our experiments (Figure 1) suggest that SCFA can be taken up across the reticulum wall effectively. In view of the absence of an electrochemical gradient in our setup, a plausible explanation for these observations is the involvement of the secondary active transport of butyrate. Nevertheless, based on our data we cannot completely exclude a contribution of diffusive pathways in the uptake of butyrate. A supportive finding for this assumption is the insensibility of J_sm_ to inhibitors and the only partial inhibition of J_ms_ under the conditions applied (Figure 3 and Figure 5). Additionally, even higher transport rates can be expected under physiological conditions—i.e., with the butyrate concentrations in the blood being 1000–2000 times lower than in the lumen of the forestomach [29,30].

Models of SCFA permeation across the rumen wall include the uptake of non-dissociated SCFA (HSCFA) from the lumen into the epithelial cells via lipophilic diffusion [4,31]. After entering the epithelium this way, HSCFA dissociate inside the cells due to their pK of ~4.8 [32], and the protons delivered by this process are extruded via NHE to maintain the intraepithelial pH [4,15,33]. Therefore, the activity of NHE may be crucial for the uptake of SCFA. In the rumen, at least the NHE isoforms 1 and 3 have been detected on a protein level [15,34]. However, the effect of NHE inhibitors on the SCFA transport across the rumen epithelium in vitro is negligible [7,35], suggesting that the export of protons out of the epithelium is of minor importance for the permeation of SCFA. In contrast, the transport of sodium is significantly reduced by the NHE inhibitors in both the rumen [15,36,37] and the reticulum epithelium [18]. Additionally, in the reticulum epithelium a reduction in the chloride flux rates was observed in the presence of NHE inhibitors [18]. In our experiments on the reticulum epithelium, neither the electrophysiological parameters nor J_ms_^but^ were affected by the application of the NHE inhibitor EIPA (Figure 2 and Figure 3). However, EIPA significantly reduced J_sm_^but^ when applied to the serosal buffer solution (Figure 3). This may hint at an involvement of NHE in J_sm_^but^. Nevertheless, the effect was small (about 10% reduction in J_sm_^but^) and observed under quite artificial conditions—i.e., 20 mmol L^−1^ butyrate in the serosal buffer solution. Therefore, it has to be questioned if NHEs play a vital role in the transport of butyrate across the reticulum epithelium under physiological conditions. However, in experiments using the same butyrate concentration on rumen epithelium the NHE inhibitors did not alter J_sm_^but^ at all [7].

Nevertheless, the coupling between SCFA transport across the rumen epithelium and sodium is evident [7,38,39]. Besides NHE, Na^+^-HCO_3_^−^ cotransporters providing bicarbonate for SCFA^−^/HCO_3_^−^ exchangers may be responsible for these observations [16]. Additionally, a co-transport of sodium and SCFA^−^ via the sodium-coupled monocarboxylate transporter 1 (SCMT1, SLC5A8) may be present [40,41]. A functional prerequisite for these mechanisms is the sodium gradient across the cell membrane established by the Na^+^/K^+^-ATPase [42,43]. In the present study on the reticulum epithelium, we therefore investigated the effect of an inhibition of the Na^+^/K^+^-ATPase by ouabain. In these experiments, the application of ouabain significantly reduced J_ms_^but^ (Figure 3), suggesting that the Na^+^/K^+^-ATPase provides a driving force that facilitates the butyrate permeation across the reticulum epithelium via the mechanisms mentioned above. In view of these data and with regard to the bicarbonate-free conditions applied in our experiments, one may speculate that, after apical uptake by lipophilic diffusion, butyrate dissociates inside the epithelium, providing protons for the apical uptake of Na^+^ via NHE. While butyrate anions may leave the epithelium via a basolateral anion channel, sodium is actively transported by the Na^+^/K^+^-ATPase, a model that is suggested for the rumen epithelium [13,31]. However, J_sm_^but^ was not affected by ouabain (Figure 3). Hence, in contrast to the absorption, the pathways for the (unphysiologic) secretion of butyrate or its metabolites seem to be independent of the activity of the Na^+^/K^+^-ATPase. Ouabain also diminished I_sc_ in our experiments (Figure 2). This is in accordance with previous findings on the same tissue [18] and was observed also in the rumen epithelium in vitro [14,44]. Besides alterations in ion distribution, the inhibition of the Na^+^/K^+^-ATPase also leads to a breakdown in the electrical gradients across the epithelium, which may have consequences for SCFA^−^ permeation as well—e.g., via anion channels [14].

The uptake of HSCFA by lipophilic diffusion and their subsequent intraepithelial dissociation would lead to an accumulation of SCFA^−^ inside the epithelium if there were not sufficient mechanisms for the extrusion of SCFA^−^ or their metabolites to the blood side [31]. One extrusion mechanism proven to be present in the rumen epithelium is proton-coupled transport via monocarboxylate transporter 1 (MCT1, SLC16A1) [10,11], which is expressed in the basolateral layer of this epithelium [34]. Another isoform, MCT4 (SLC16A3), is expressed on the apical side of the rumen epithelium and could act in concert with MCT1 in the transport of SCFA from the lumen to the blood [45]. At least MCT1 has been shown to be expressed also in the basolateral layers of the reticulum epithelium [46]. Therefore, we investigated the effect of an MCT inhibitor in this tissue.

In in vitro studies on the rumen epithelium, pHMB has been used as an inhibitor of MCTs and it reduced the J_ms_ of acetate but had no effect on J_ms_^but^ [12]. In another study on the rumen epithelium, it significantly reduced the serosal-to-mucosal flux of acetate [47]. In our study on the reticulum epithelium, pHMB significantly reduced the J_ms_^but^ but did not affect the flux rates in the opposite direction (Figure 5). The reduction in J_ms_^but^ was observed after both the mucosal and serosal application of pHMB. Hence, the effect of pHMB on the flux rates of SCFA across the reticulum epithelium is not congruent to that observed on the rumen epithelium.

To explain our results, one may speculate that, in addition to the basolateral MCT1 [46], an apical MCT is expressed in the reticulum epithelium, similar to the rumen epithelium [45]. Furthermore, one could assume that the apical MCT prefers butyrate as a substrate while the basolateral MCT1 could have a preference for its metabolites. Hints as to differences between MCT1 and 4 have been found in the intestinal epithelial cell line IEC-18 [48]. Based on this, apically applied pHMB would reduce the apical uptake of butyrate (J_ms_^but^) but would not affect the apical extrusion of the metabolites (as indicated by J_sm_^but^). On the other hand, serosally applied pHMB would reduce the basolateral extrusion of the metabolites (J_ms_^but^) but would have no effect on the basolateral uptake of butyrate (J_sm_^but^).

However, besides the reduction in J_ms_^but^, pHMB strikingly affected G_t_ and I_sc_ in our experiments (Figure 4), suggesting that the reduction in J_ms_^but^ observed after the pHMB addition may not be solely attributed to an inhibition of the “electrically silent” MCTs.

As a thiol reagent, pHMB may influence the activity and/or structure of a plethora of proteins [49], resulting in functional alterations of the epithelium.

Hence, the increase in the G_t_ after both the mucosal and serosal application of pHMB (Figure 4), indicating a reduced tightness of the epithelium, might be a result of tight junction modifications. Likewise, the increase in the I_sc_ after the addition of pHMB to the mucosal buffer solution (Figure 4) may be a result of the modification of apical cation channels, as proved to be present in the rumen epithelium [50]. Additionally, pHMB inhibits various ATPases by interacting with the SH-groups of the enzyme [51,52]. Therefore, the serosal application of pHMB may inhibit the Na^+^/K^+^-ATPase rather than MCT1. This could explain why the decrease in the I_sc_ observed in our studies after the serosal addition of pHMB shows a similar course and extent as after ouabain treatment (Figure 2 and Figure 4). Additionally, the reduction in J_ms_^but^ after the application of pHMB to the serosal buffer solution is similar to that after ouabain application (Figure 3 and Figure 5). Additionally, the reduction in J_ms_^but^ after the addition of pHMB to the serosal side could be attributed to the inhibition of a basolateral anion channel permeable for butyrate or its metabolites [53]. Since the permeation via such a channel is energized by the electrical gradient [13], J_sm_^but^ would occur against this gradient and might therefore be small and insensitive to pHMB (Figure 5). However, a channel-mediated permeation of butyrate is not reflected in our electrophysiological data (Figure 4).

Our experiments cannot unravel this completely, but future studies might contribute by applying more specific inhibitors. Still, our study succeeds in gaining a first impression on the permeation of SCFA across the reticulum epithelium.

Our investigations focused on bicarbonate-independent mechanisms, knowing full well that the exchange of SCFA^−^ for bicarbonate is one of the main uptake pathways for SCFA across the rumen epithelium [9,12,54]. In this regard, it has also to be mentioned that MCT1 may work as a HCO_3_^−^/SCFA^−^ exchanger under certain conditions [12]. We assume that bicarbonate-dependent pathways contribute substantially to the SCFA permeation across the reticulum epithelium as well, though differences to the rumen may become apparent in detail.

## 5. Conclusions

Our study clearly demonstrates that butyrate is taken up effectively across the reticulum epithelium in a secondary active transport mechanism energized by Na^+^/K^+^-ATPase. This is indicated by the absolute unidirectional flux rates, the positive net flux, and the inhibition of the butyrate flux rates by ouabain. However, the results of the experiments using the inhibitors EIPA or pHMB differ from observations made using these compounds on the rumen epithelium. Hence, it can be assumed that the mechanisms involved in the SCFA permeation across the reticulum epithelium are not completely congruent to those in the rumen epithelium. These modifications may assure the effective uptake of the energy substrates from the reticulum lumen under the particular conditions of this forestomach compartment.

## Figures and Tables

**Table 1:** Statistical metrics of the electrophysiological parameters I_sc_ and G_t_ regarding the effects of pHMB. Differences (Δ) were calculated as mean 2nd hour (i.e., with inhibitor) mean 1st hour (i.e., without inhibitor). *p* is given as the result of a paired Student’s t-test versus control, asterisks indicate statistically significant differences. Mean ± SD, N = 6.

Metric	Control	Mucosal pHMB	Serosal pHMB
I_sc_ mean, 1st flux period	2.37 ± 0.49	2.64 ± 0.62 *^p^*^ = 0.118^	2.67 ± 0.68 *^p^*^ = 0.106^
I_sc_ mean, 2nd flux period	2.04 ± 0.58	2.9 ± 0.76 * *^p^*^ = 0.007^	0.54 ± 0.28 * *^p^*^ = 0.003^
Δ I_sc_ mean	−0.34 ± 0.17	0.26 ± 0.38 * *^p^*^ = 0.005^	−2.13 ± 0.59 * *^p^*^ = 0.001^
Max I_sc_	2.49 ± 0.49	5.04 ± 2.05 * *^p^*^ = 0.012^	2.75 ± 0.68 *^p^*^ = 0.13^
Min I_sc_	1.82 ± 0.45	2.44 ± 0.55 * *^p^*^ = 0.006^	0.39 ± 0.27 * *^p^*^ = 0.001^
G_t_ mean, 1st flux period	4.83 ± 2.97	4.85 ± 1.83 *^p^*^ = 0.988^	4.31 ± 1.01 *^p^*^ = 0.652^
G_t_ mean, 2nd flux period	4.78 ± 3.05	6.34 ± 2.51 *^p^*^ = 0.363^	6.22 ± 1.46 *^p^*^ = 0.21^
Δ G_t_ mean	−0.04 ± 0.16	1.51 ± 0.73 * *^p^*^ = 0.004^	1.91 ± 0.72 * *^p^*^ = 0.000^
Max G_t_	5.64 ± 3.62	6.58 ± 2.66 *^p^*^ = 0.623^	7.00 ± 1.76 *^p^*^ = 0.322^
Min G_t_	4.56 ± 2.85	4.76 ± 1.82 *^p^*^ = 0.891^	4.2 ± 0.99 *^p^*^ = 0.752^
